# A model of type 2 diabetes in the guinea pig using sequential diet-induced glucose intolerance and streptozotocin treatment

**DOI:** 10.1242/dmm.025593

**Published:** 2017-02-01

**Authors:** Brendan K. Podell, David F. Ackart, Michael A. Richardson, James E. DiLisio, Bruce Pulford, Randall J. Basaraba

**Affiliations:** Department of Microbiology, Immunology and Pathology, College of Veterinary Medicine and Biomedical Sciences, Colorado State University, Fort Collins, CO 80523, USA

**Keywords:** Animal model, Insulin-independent diabetes, Guinea pig, Glucose intolerance, Streptozotocin, Type 2 diabetes

## Abstract

Type 2 diabetes is a leading cause of morbidity and mortality among noncommunicable diseases, and additional animal models that more closely replicate the pathogenesis of human type 2 diabetes are needed. The goal of this study was to develop a model of type 2 diabetes in guinea pigs, in which diet-induced glucose intolerance precedes β-cell cytotoxicity, two processes that are crucial to the development of human type 2 diabetes. Guinea pigs developed impaired glucose tolerance after 8 weeks of feeding on a high-fat, high-carbohydrate diet, as determined by oral glucose challenge. Diet-induced glucose intolerance was accompanied by β-cell hyperplasia, compensatory hyperinsulinemia, and dyslipidemia with hepatocellular steatosis. Streptozotocin (STZ) treatment alone was ineffective at inducing diabetic hyperglycemia in guinea pigs, which failed to develop sustained glucose intolerance or fasting hyperglycemia and returned to euglycemia within 21 days after treatment. However, when high-fat, high-carbohydrate diet-fed guinea pigs were treated with STZ, glucose intolerance and fasting hyperglycemia persisted beyond 21 days post-STZ treatment. Guinea pigs with diet-induced glucose intolerance subsequently treated with STZ demonstrated an insulin-secretory capacity consistent with insulin-independent diabetes. This insulin-independent state was confirmed by response to oral antihyperglycemic drugs, metformin and glipizide, which resolved glucose intolerance and extended survival compared with guinea pigs with uncontrolled diabetes. In this study, we have developed a model of sequential glucose intolerance and β-cell loss, through high-fat, high-carbohydrate diet and extensive optimization of STZ treatment in the guinea pig, which closely resembles human type 2 diabetes. This model will prove useful in the study of insulin-independent diabetes pathogenesis with or without comorbidities, where the guinea pig serves as a relevant model species.

## Abstract (precis)

**Summary:** The guinea pig can act as a tool for investigators interested in the translational manifestations of diabetes using new methods for induction of type 2 diabetes.

## INTRODUCTION

Among noncommunicable diseases, type 2 diabetes is a major cause of morbidity and mortality worldwide. Among the different forms of diabetes, type 2 diabetes accounts for more than 90% of the diabetic population, and the prevalence is rapidly increasing on a global scale with an estimated increase from 387 million diabetic patients in 2014 to ∼600 million by 2035 (Guariguata et al., 2014; IDF, 2015). Of particular importance is the recent increase in the incidence of type 2 diabetes among low- and middle-income countries, which also have the highest population of prediabetic patients worldwide (Guariguata et al., 2014; IDF, 2015). In remote global regions, ∼50% of the diabetic population is undiagnosed and therefore is living with uncontrolled diabetes (Beagley et al., 2014; IDF, 2015). Moreover, this increase in the incidence of diabetes in low- and middle-income countries highlights a new convergence of noncommunicable diseases with endemic communicable diseases, thereby complicating the global control of important infectious diseases as well (Remais et al., 2013). Accordingly, identification of preventive and therapeutic measures that are affordable and applicable to populations in low- and middle-income countries will be paramount to controlling the growing epidemic of type 2 diabetes. The development and testing of new prevention and treatment strategies is dependent on their initial preclinical evaluation using animal models that mimic the pathogenesis of the human disease.

Type 2 diabetes is a complex metabolic disorder that results from chronic inflammation-associated alterations in lipid metabolism and corresponding insensitivity of tissues to the actions of insulin. Obesity, the most common risk factor for the development of type 2 diabetes, links the accumulation and inflammation of excess adipose tissue with alterations in systemic lipid metabolism and the development of impaired glucose homeostasis. This link in metabolic disturbances has been termed metabolic syndrome and represents a combination of elevated serum triglycerides, hypertension and evidence of insulin resistance, all of which contribute to the pathogenesis of type 2 diabetes and diabetes-associated complications (Shoelson et al., 2006). Owing to the complexity of this disease, preclinical animal models used to study complications, comorbidities or therapeutic interventions for type 2 diabetes are essential and must accurately reflect the pathogenesis of the human disease.

A number of animal models of insulin-independent type 2 diabetes and insulin-dependent diabetes exist, but their application in comparative biomedical research depends on the context of the disease being studied. Experimentally induced high-fat diets are commonly used in inbred mouse or rat strains to model metabolic syndrome and the associated adiposity, dyslipidemia and cardiovascular disease phenotypes (Srinivasan and Ramarao, 2007; Surwit et al., 1988). However, these models require lengthy feeding regimens before any decline in β-cell mass is detectable. To overcome this barrier, streptozotocin (STZ) has been described in the rat to deplete β-cell mass experimentally after induction of diet-induced insulin resistance (Reed et al., 2000; Islam and Loots, 2009; Islam and Wilson, 2012; Skovsø, 2014). This experimental approach might best model the natural pathogenesis of overt type 2 diabetes in humans. Guinea pigs might offer additional advantages over existing rodent models of type 2 diabetes and metabolic syndrome. Although guinea pigs have been previously used as a diabetes model only through induction with STZ, this serves specifically as a model of insulin-dependent diabetes because it lacks the contribution of insulin resistance. Moreover, there are substantial differences published in the overall susceptibility of the guinea pig to chemical induction of diabetes with STZ, which are the consequence of a wide range of experimental approaches (Wehner and Majorek, 1975; Klein et al., 1980; Schlosser et al., 1984, 1987; Aomine et al., 1990; Lenzen, 2007).

Although the guinea pig is not expected to replace more common mouse or rat rodent models of type 2 diabetes, this species does have certain advantages. In addition to inflammatory changes induced by high-fat and high-sugar diets (Fernandez and Volek, 2006; Ye et al., 2013), the guinea pig is widely regarded for research in specific diseases, including cardiovascular disease, atherosclerosis and arthritis, as well as a number of infectious diseases that have been linked as comorbidities with diabetes (West and Fernandez, 2004; Madsen et al., 2008; Padilla-Carlin et al., 2008). The guinea pig is crucial for development of new vaccines, particularly because of its immunological and pathological similarities in response to a number of infectious diseases of humans (Hickey, 2011). Additionally, the guinea pig, more so than any other rodent, shares commonalities with human lipid metabolism, including cholesterol metabolism and transport, with a greater proportion of cholesterol carried in association with low-density lipoproteins (Ensign et al., 2002; Fernandez et al., 1999; Ye et al., 2013).

Given the significant variability and reported resistance of guinea pigs to experimental diabetogenic treatments, we investigated the impact of a high-fat, high-carbohydrate (HFHC) diet on glucose tolerance and induction of insulin resistance, and the impact of pre-existing glucose intolerance on induction of overt diabetes with STZ. Herein, we have developed a representative model of human insulin resistance and type 2 diabetes in the guinea pig by strategically promoting the sequential development of glucose intolerance with HFHC diet, followed by β-cell loss through partial cytotoxicity. This experimental induction of increased insulin demand along with a reduction in functional β-cell mass is designed to mimic crucial manifestations in the pathogenesis of human type 2 diabetes, which can be accomplished within a condensed time frame (Kahn, 2001; Ferrannini et al., 2005; Weir and Bonner-Weir, 2013).

## RESULTS

### HFHC-fed guinea pigs develop dyslipidemia in the absence of rapid weight gain

Weight change was monitored over time in guinea pigs fed either a normal guinea-pig diet or the HFHC diet, then for 3 weeks following STZ treatment of HFHC-fed guinea pigs (Fig. S1). Guinea pigs fed either normal diet or HFHC diet had comparable caloric intake based on body weight (Table S1), and after 8 weeks of consuming the HFHC diet, control guinea pigs fed a normal diet weighed a mean 700.9±48.8 g, compared with 571.1±45.2 g for HFHC-fed guinea pigs (*P*<0.05). This difference was the result of initial weight loss and subsequent static weight gain during the transition to the HFHC diet. After this transition period, HFHC-fed guinea pigs demonstrated a similar mean rate of weight gain (2.75±0.17 g/day) compared with normal-diet controls (2.38±0.15 g/day; Fig. S1). However, HFHC/STZ guinea pigs with diabetic glucose intolerance had significant weight loss (*P*<0.001) over the 3 week period following STZ administration, with a mean weight of 565.2±45.2 g before STZ treatment and 441.6±42.4 g 3 weeks after STZ. Both HFHC-fed and HFHC/STZ guinea pigs had elevated fasting serum triglyceride concentrations compared with normal-diet controls; however, elevations in total serum free fatty acids were present only in the HFHC/STZ guinea pigs (Table 1). Hepatocellular triglycerides were also elevated in HFHC/STZ guinea pigs; however, in response to oral antihyperglycemic therapy with metformin and glipizide in combination, concentrations of hepatocellular triglycerides were significantly lowered compared with HFHC/STZ guinea pigs with uncontrolled diabetes (Table 1). Most of the cholesterol in all treatment groups was contained in low-density lipoprotein (LDL)/very low-density lipoprotein (VLDL) serum fractions, as previously reported, but HFHC feeding, both with and without STZ, significantly increased LDL/VLDL cholesterol compared with normal-diet controls without altering HDL cholesterol (Table 1).

**Table 1.**
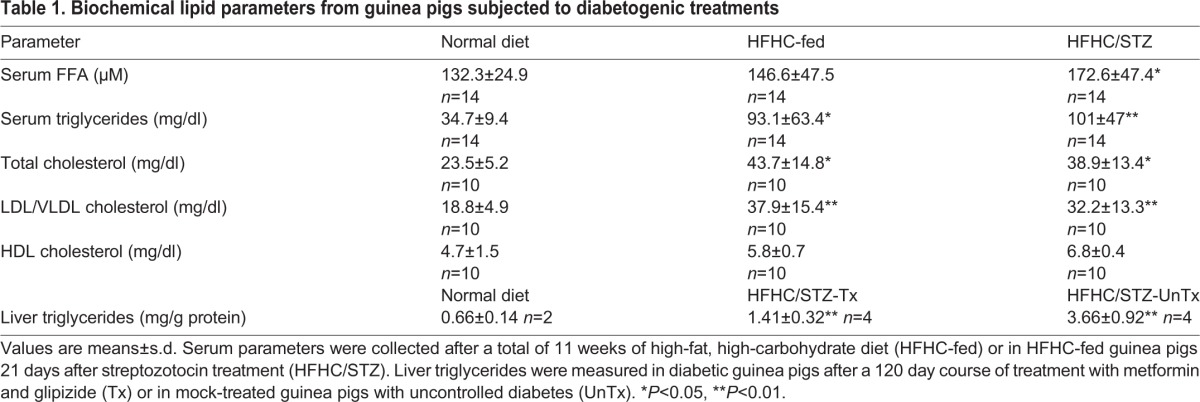
**Biochemical lipid parameters from guinea pigs subjected to diabetogenic treatments**

### HFHC-fed guinea pigs develop impaired glucose tolerance and a suppressed response to exogenous insulin

**Fig. 1.**
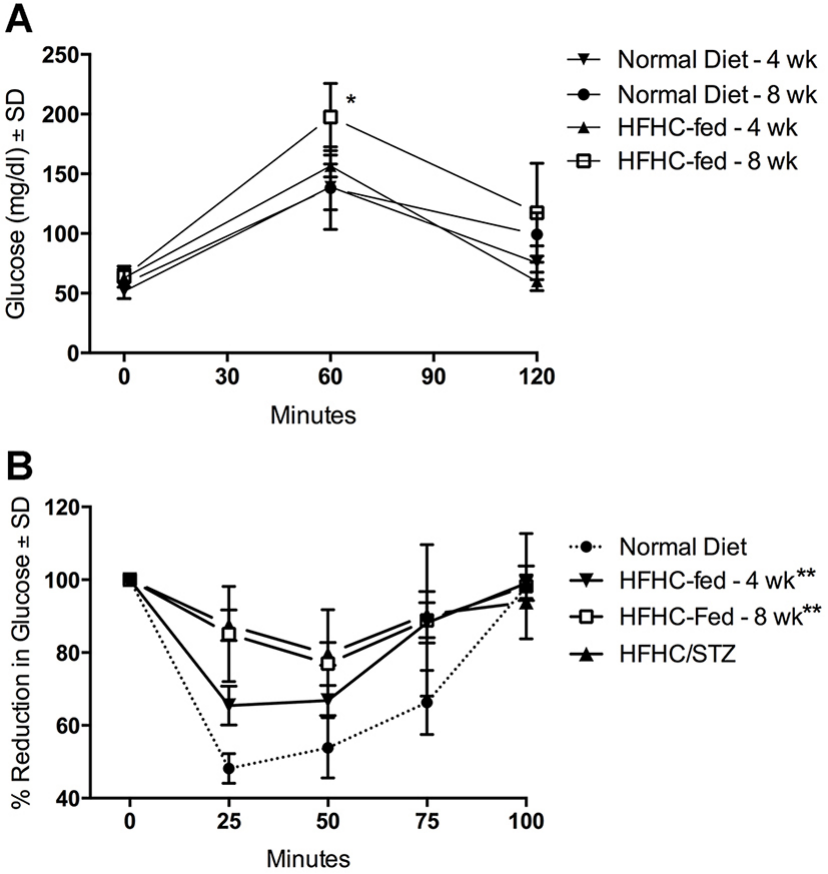
**Guinea pigs fed HFHC diet develop impaired glucose tolerance and suppressed response to insulin.** Fasted or nonfasted guinea pigs were challenged with an oral glucose bolus or exogenous insulin, respectively, and blood glucose was measured over time. (A) Response to an oral glucose challenge in guinea pigs that consumed HFHC diet for 4 or 8 weeks (*n*=20), compared with normal-diet controls (*n*=20). Fasting hyperglycemia is absent, but delayed glucose clearance is evident 60 min after glucose challenge in HFHC-fed guinea pigs. *P<0.05, two-way ANOVA. (B) Response of HFHC-fed guinea pigs to an insulin tolerance test (*n*=5) after 4 or 8 weeks of HFHC feeding or in HFHC/STZ guinea pigs compared with normal-diet controls (*n*=5). Impaired tissue sensitivity to insulin is apparent in HFHC-fed and HFHC/STZ guinea pigs, based on failure to reduce blood glucose over time compared with normal-diet controls. **P<0.01, based on mean area under the curve, Student's *t*-test.

HFHC-fed guinea pigs were evaluated for evidence of glucose intolerance based on an oral glucose tolerance test (OGTT). No significant difference in the glucose response was observed between normal-diet controls and HFHC-fed guinea pigs after 4 weeks on the diet (Fig. 1A). However, after 8 weeks of consuming the HFHC diet, impaired glucose tolerance was evident as increased average blood glucose at the 60 min time point of the OGTT (197.5±28.3 versus 138±34.6 mg/dl; Fig. 1A). Significantly elevated fasting blood glucose was not ever observed in guinea pigs fed HFHC diet alone. To identify further evidence of insulin resistance, the reduction in blood glucose was measured in HFHC-fed guinea pigs in response to exogenous insulin. Exogenous insulin treatment induced a rapid reduction in blood glucose within 30 min in normal-diet control guinea pigs, with a gradual return to euglycemia over 100 min. Although no difference was seen at 4 weeks of HFHC feeding based on the OGTT, the response to exogenous insulin was suppressed after 4 weeks and significantly impaired after a total of 8 weeks of HFHC feeding (Fig. 1B).

### Optimized STZ dosing minimizes toxicity-associated mortality in the guinea-pig model

In developing a guinea-pig model of STZ-induced hyperglycemia, three parameters were identified as being crucial for a successful response to STZ without adverse effects, including the dose, route and preparation of the STZ solution (Table 2, Fig. S2). As multiple doses, routes of administration and methods for preparation have been previously reported in guinea pigs or other rodent species, an encompassing approach to optimization was taken for this guinea-pig model using guinea pigs fed a normal diet. In guinea pigs receiving 300 mg/kg, regardless of whether the subcutaneous (SC) or intraperitoneal (IP) route was used, or single versus multiple dosing, a high level of mortality occurred within days after completing the treatment. Mortality in guinea pigs dosed with STZ 300 mg/kg was 80% in those receiving a single dose by the SC route (*n*=5), 75% in those receiving a single dose by the IP route (*n*=4) and 100% in those receiving six consecutive daily doses of 50 mg/kg IP (*n*=4), using a multidose schedule shown to be effective in mice (Qi et al., 2005). Only one of 13 guinea pigs receiving a cumulative 300 mg/kg dose ever developed evidence of hyperglycemia.

**Table 2.**
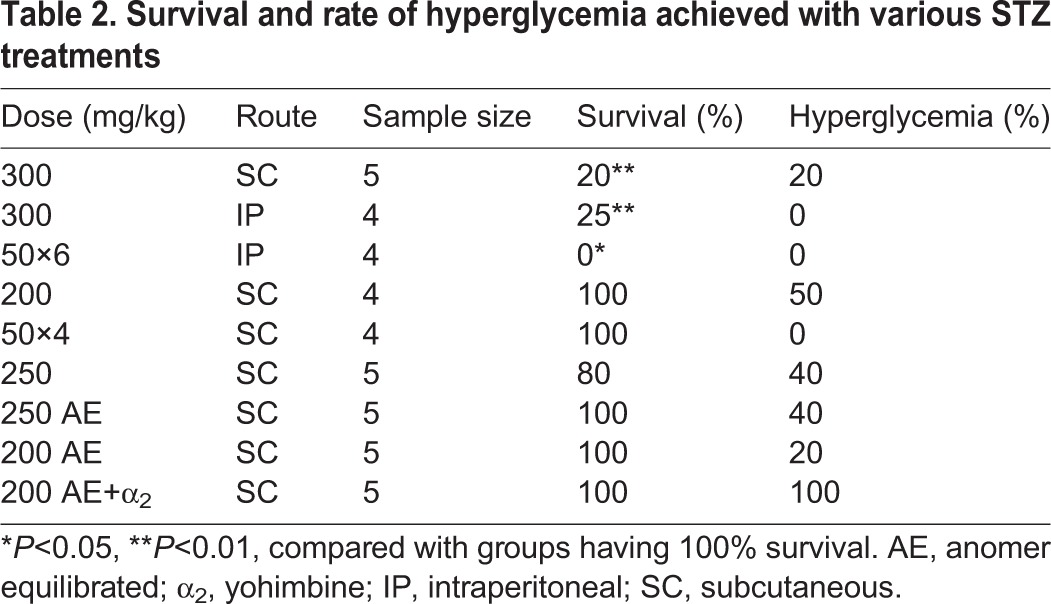
**Survival and rate of hyperglycemia achieved with various STZ treatments**

Evaluation of histopathology revealed that mortality in guinea pigs was the result of acute STZ-mediated toxicity. The most severe and frequent manifestation of acute STZ toxicity was acute necrosis of the intestinal mucosal epithelium, followed by acute necrosis of renal tubular epithelium (Fig. S3). There were no differences in toxicity-related pathology in the small intestine and kidney related to the route of administration or frequency of dosing in those receiving a total of 300 mg/kg, and similar lesions in these organs were associated with mortality after lower doses of STZ. Although no significant differences in mortality were observed between IP and SC routes of administration, guinea pigs that received IP STZ developed mild peritonitis and exuberant peritoneal and serosal fibrosis on visceral organs, precluding further use of this route of administration (Fig. S3).

**Fig. 2.**
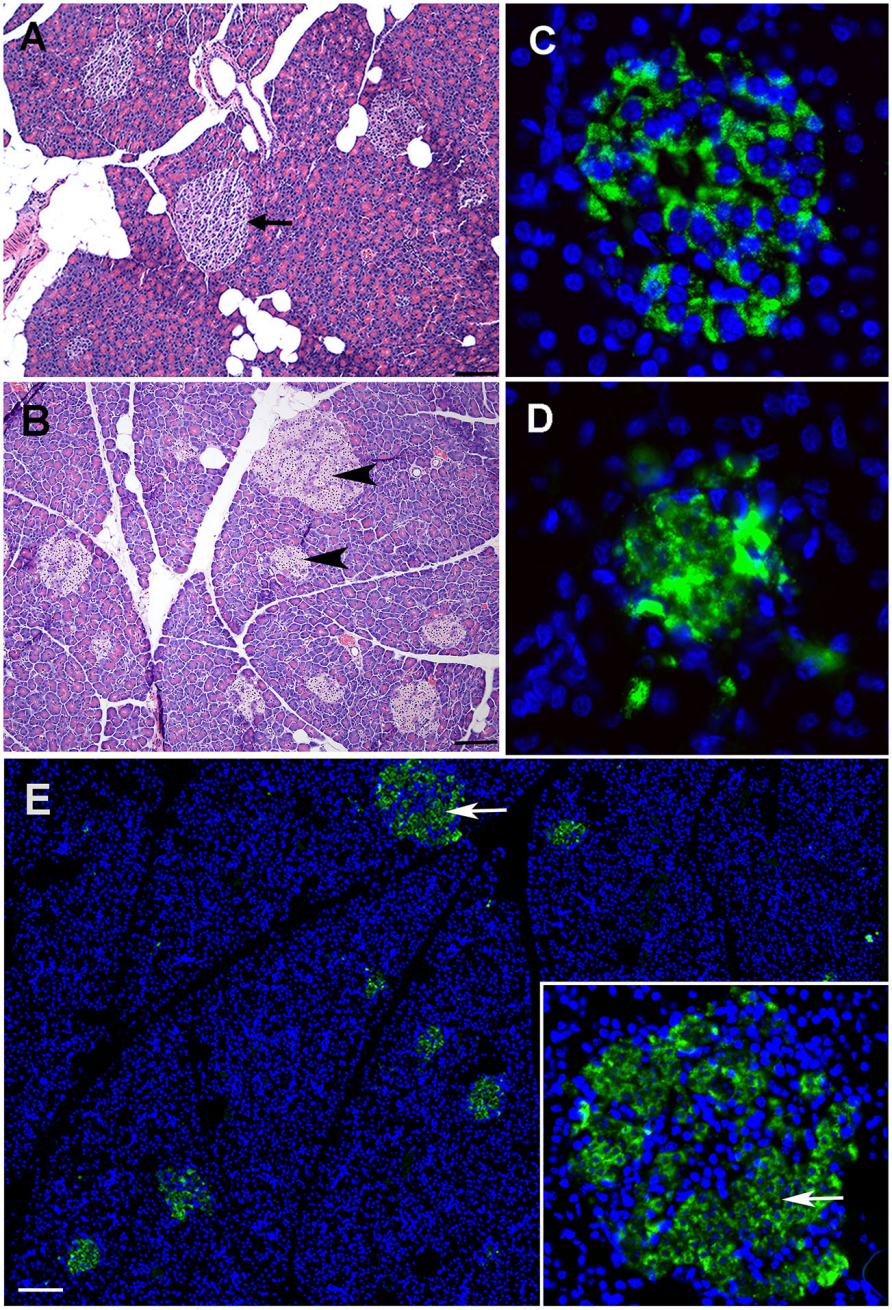
**Optimized STZ treatment leads to specific cytotoxicity of insulin-producing cells.** Images are representative of guinea pigs developing hyperglycemia after treatment with an optimized SC dose of anomer-equilibrated STZ at 200 mg/kg administered after an intramuscular injection of yohimbine. (A) Pancreatic islets from a nondiabetic, normal-diet control guinea pig (arrow) demonstrating histological morphology in the absence of diabetogenic treatment. (B) Morphological changes 48 h after STZ treatment in pancreas of a guinea pig with confirmed hyperglycemia that was fed a normal diet. Cell death has occurred in the majority of islet cells (arrowheads), while a minority of the cells remain viable. (C) Immunofluorescent detection of pro-insulin (green) in pancreatic islets in a normal-diet control guinea pig indicates that the majority of islet cells are insulin-producing β cells (blue, Hoechst nuclear counterstain). (D,E) Immunofluorescent detection of pro-insulin in a guinea pig 48 h after receiving an optimized dose of STZ. Disruption of cellular and nuclear morphology is uniform across all islets and is specific to insulin-expressing β cells (arrows and inset). Scale bar: 100 µm.

By contrast, improved STZ tolerance and survival was achieved with 200 mg/kg as a single- or multiple-dose schedule, with 100% survival to 30 days post-injection (Table 2). However, the hyperglycemia response rate was unacceptably low at this dose, with no induction at four doses of 50 mg/kg (*n*=4) and only 50% with a single dose (*n*=4). This rate of response was not improved upon by a slightly higher single dose of STZ, 250 mg/kg (Table 2). There was no histological evidence of β-cell death within pancreatic islets in guinea pigs treated with a 300 mg/kg dose. However, acute cell death was evident within the islets of guinea pigs treated with 250 mg/kg doses of STZ (Fig. 2B) and corresponded to aggregates of insulin protein, presumably released from β-cell necrosis (Fig. 2D).

As it is well demonstrated that the α-anomeric form of STZ is more likely to induce adverse toxicity, we evaluated the preparation of STZ as an equilibrated solution of α- and β-anomers to determine whether, at an equivalent dose, the equilibrated solution of STZ is capable of producing a similar hyperglycemic response (Bell and Hye, 1983; de la Garza-Rodea et al., 2010). At a dose of 250 mg/kg, both freshly prepared and anomer-equilibrated STZ induced nonfasting hyperglycemia in 40% of treated guinea pigs, indicating a similar efficacy between both preparations (Table 2). Guinea pigs receiving anomer-equilibrated STZ demonstrated 100% survival 21 days after STZ treatment, compared with 80% survival in guinea pigs receiving freshly prepared STZ, after which the experiment was terminated.

### STZ-induced hyperglycemia is enhanced with antecedent use of an α_2_-adrenergic receptor antagonist

As the success rate of achieving hyperglycemia in guinea pigs at mid-range doses of STZ remained low regardless of the dose or route of administration (Table 2), the α_2_-antagonist yohimbine was evaluated as an adjunctive treatment, because it has been demonstrated to enhance the diabetogenic effects of STZ in mice (Nakadate et al., 1981). We evaluated the rate of hyperglycemia from anomer-equilibrated STZ treatment in guinea pigs either pretreated with yohimbine or mock-treated with normal saline. Guinea pigs that were pretreated with yohimbine were 100% responsive to the STZ treatment as indicated by hyperglycemia present for the following 7 day period (Table 2). By contrast, 20% of the mock-treated guinea pigs developed hyperglycemia.

### STZ treatment leads to sustained diabetic glucose intolerance only in the presence of subclinical impaired glucose tolerance attributable to HFHC feeding

**Fig. 3.**
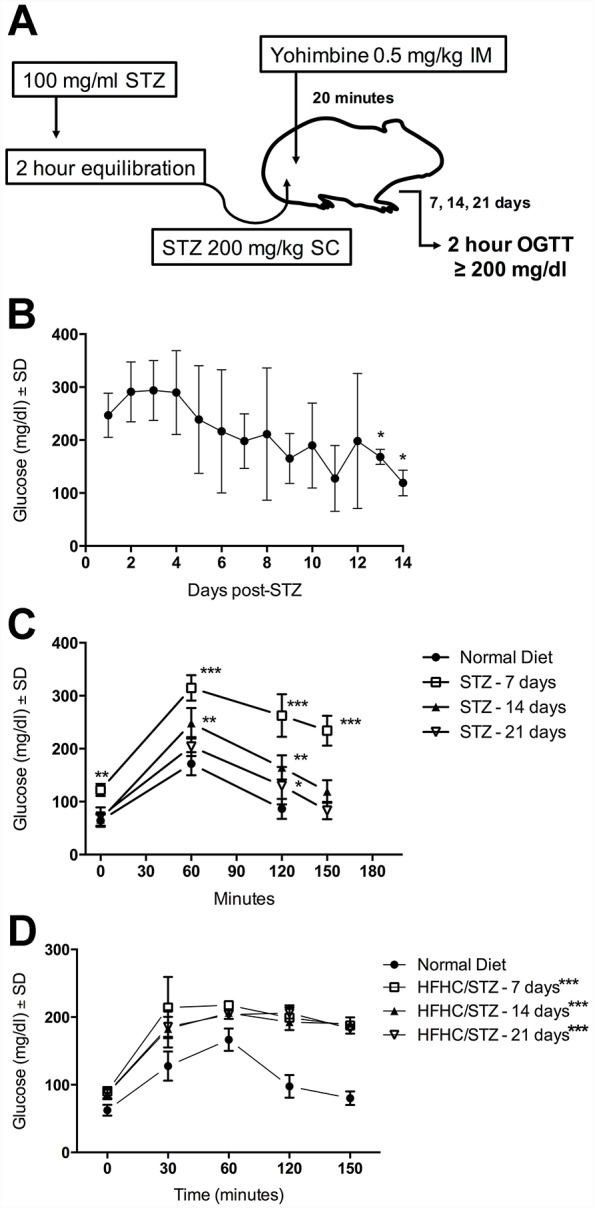
**Development of stable STZ-induced diabetes requires coexisting diet-induced impaired glucose tolerance.** (A) Diagrammatic representation of optimized STZ treatment to induce hyperglycemia in guinea pigs fed either normal or HFHC diet. STZ powder is dissolved in citrate buffer at 100 mg/ml, then incubated for 2 h to allow for α/β anomer equilibration. Twenty minutes before STZ treatment, guinea pigs are administered a 0.5 mg/kg dose of the α_2_ agonist yohimbine, by the intramuscular (IM) route. Guinea pigs are then given a single 200 mg/kg subcutaneous (SC) injection of anomer-equilibrated STZ. Diabetic hyperglycemia is determined by an oral glucose tolerance test (OGTT) at day 7, 14 and 21, based on a 2 h blood glucose ≥200 mg/dl. (B) Blood glucose concentrations were measured daily at random in nonfasted, STZ-treated guinea pigs fed a normal diet (*n*=5). Hyperglycemia, observable 24 h after STZ treatment, has a trend of steady decline over the course of 14 days. *P<0.05 compared with day 1 post-STZ, one-way ANOVA. (C) Glucose tolerance was evaluated in STZ-treated guinea pigs (*n*=5) fed a normal diet by standardized oral glucose challenge on day 7, 14 and 21 after STZ treatment, and compared with normal-diet controls (*n*=5). Fasting hyperglycemia and diabetic glucose intolerance is evident 7 days after STZ treatment, but glucose intolerance diminishes over time, returning to nondiabetic tolerance after 21 days. **P<0.01, ***P<0.001, compared with mean glucose of normal-diet controls at 0, 60 and 120 min, or day 21 challenge of STZ guinea pigs at 150 min, two-way ANOVA. (D) HFHC-fed guinea pigs were treated with STZ after 8 weeks of consuming HFHC diet. Response to an oral glucose challenge is documented over 7, 14 and 21 days after STZ treatment that was preceded by 8 weeks of HFHC feeding (*n*=5 or 10). STZ treatment results in stable fasting hyperglycemia and diabetic glucose intolerance persisting to 21 days after treatment when guinea pigs are challenged in the presence of HFHC diet-induced impaired glucose tolerance. *P<0.05, ***P<0.001, compared with mean glucose of normal-diet controls at that measurement time point, one-way ANOVA based on mean area under the curve.

At STZ doses of 200 and 250 mg/kg (with or without yohimbine), where hyperglycemia was manifested in 25-100% of treated guinea pigs, elevated glucose was evident within 48 h. During daily random glucose sampling, nonfasted hyperglycemia persisted in the range of 200-400 mg/dl, for 7-10 days. There was a consistent steady decline in the degree of hyperglycemia, beginning at ∼day 7 and extending to day 21 post-STZ treatment. By day 14 post-STZ treatment, random glucose samples were often within the normal range of untreated, nondiabetic guinea pigs (Fig. 3B). This transient response was uniform across all guinea pigs that developed hyperglycemia within 7 days of STZ treatment. Evaluation of glucose tolerance by OGTT in guinea pigs treated with STZ alone after 14 and 21 days demonstrated that glucose tolerance returned to normal over time, when compared with an OGTT performed on day 7 after treatment, when severe glucose intolerance was present (Fig. 3C). By contrast, guinea pigs initially fed the HFHC diet for 8 weeks to induce glucose intolerance, then treated with yohimbine and 200 mg/kg of STZ (HFHC/STZ), developed persistent diabetic levels of glucose intolerance and fasting hyperglycemia based on human diagnostic criteria. On day 21 post-STZ treatment, HFHC/STZ guinea pigs continued to show fasting hyperglycemia, a feature that was absent from HFHC-fed guinea pigs. In response to oral glucose challenge, HFHC/STZ guinea pigs demonstrated severely impaired glucose tolerance, with elevated glucose exceeding 200 mg/dl after 2 h, which persisted after 21 days post-STZ treatment (Fig. 3D).

### HFHC-fed guinea pigs display compensatory hyperinsulinemia, which is eliminated in HFHC/STZ-treated guinea pigs

**Fig. 4.**
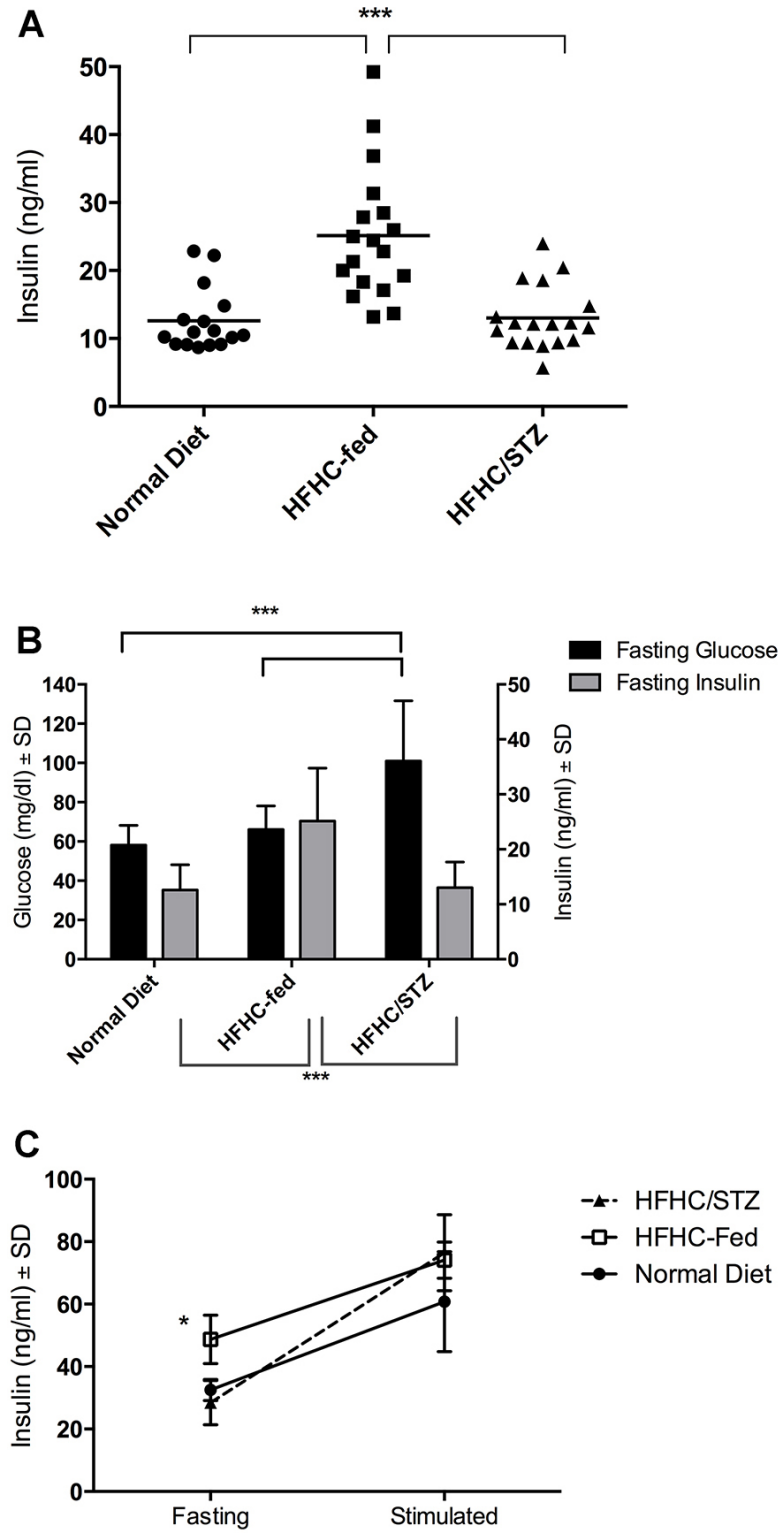
**Compensatory hyperinsulinemia maintains euglycemia in HFHC-fed guinea pigs but is lost with STZ treatment.** (A) Insulin was detected by direct ELISA in serum from fasted guinea pigs fed a normal diet (*n*=16), HFHC diet (*n*=18) or HFHC diet and treated with STZ (HFHC/STZ, *n*=18). Fasting hyperinsulinemia is present in guinea pigs after 8 weeks of consuming the HFHC diet. Treatment of HFHC-fed guinea pigs with STZ leads to impairment of the compensatory insulin response; however, insulin production is retained in HFHC/STZ guinea pigs at a level comparable to normal-diet controls. ***P<0.001, one-way ANOVA. (B) Fasting blood glucose concentrations were measured from fasting samples taken simultaneously for insulin quantification by direct ELISA. Fasting hyperglycemia is absent in the presence of compensatory hyperinsulinemia in HFHC-fed guinea pigs. Fasting hyperglycemia is present in HFHC/STZ guinea pigs after impairment of the compensatory insulin response. ***P<0.001, one-way ANOVA. (C) Glucose-stimulated insulin secretion performed in guinea pigs after 8 weeks of HFHC feeding, then repeated 21 days after receiving STZ. Residual function is retained in β cells after STZ treatment (*n*=3). *P<0.05, paired one-way ANOVA.

Fasted serum insulin concentrations were compared along with fasting blood glucose from the same blood sample to determine whether a compensatory response was present in HFHC-fed and HFHC/STZ guinea pigs (Fig. 4A). Compared with normal-diet controls, mean fasting insulin concentrations were 2.1-fold higher in HFHC-fed guinea pigs after consuming the diet for 8 weeks; however, fasting glucose concentrations did not differ from those of normal-diet controls. In HFHC/STZ guinea pigs 21 days after STZ-treatment, serum insulin concentrations were comparable to normal-diet control guinea pigs, at 13.0±4.7 and 12.6±4.6 ng/ml, respectively. However, fasting glucose concentrations of HFHC/STZ guinea pigs were significantly elevated over normal-diet controls at 101.0±30.6 and 58.1±10.1 mg/dl, respectively (Fig. 4B). To determine the insulin-secretory capacity in response to glucose challenge, fasting insulin concentrations were paired with stimulated samples 30 min after an oral glucose bolus. Although fasting insulin concentrations were decreased in HFHC/STZ guinea pigs, circulating insulin increased to comparable concentrations after 30 min, indicating that when stimulated, residual insulin-secretory capacity exists after STZ treatment (Fig. 4C).

### Islet cell hyperplasia accompanies HFHC-induced hyperinsulinemia and residual insulin production in islets of HFHC/STZ-treated guinea pigs

**Fig. 5.**
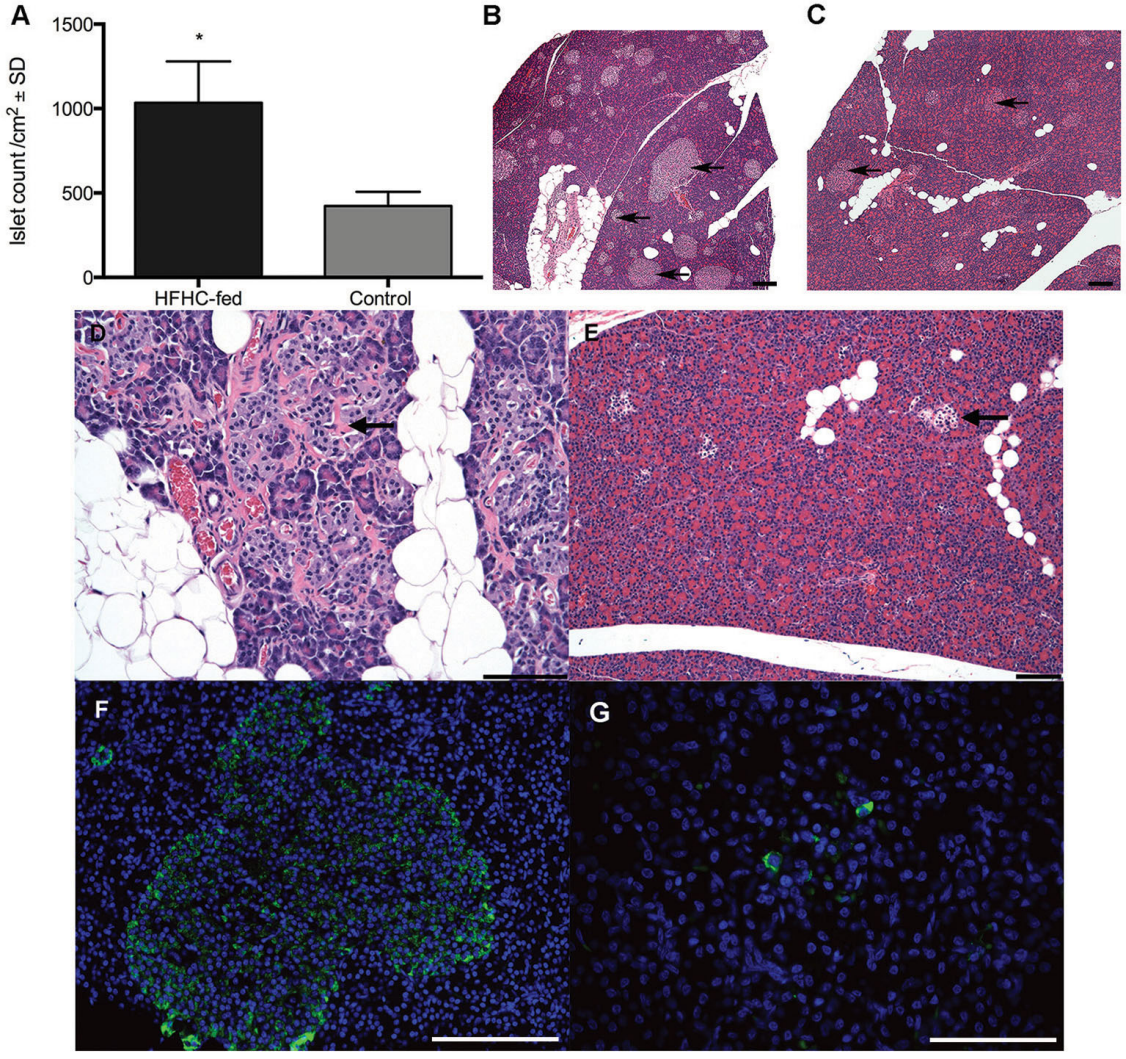
**Morphological alterations in pancreatic islets demonstrate a compensatory response to HFHC diet and STZ-induced β-cell loss.** (A) Pancreas tissue area was quantified using morphometry software and frequency of islets expressed per centimeter squared of total pancreatic tissue (*n*=5 per group). HFHC-fed guinea pigs develop islet hyperplasia after 8 weeks of consuming HFHC diet, consistent with insulin resistance and a compensatory response. *P<0.05, Student's *t*-test. (B) Histological morphology of pancreas from an HFHC-fed guinea pig. Islets are both enlarged and more frequent compared with normal-diet controls (arrows). (C) Pancreas from a normal-diet control guinea pig demonstrates the typical frequency and size of pancreatic islets in the absence of diabetogenic treatment (arrows). Scale bar: 100 µm. (D) Representative islet morphology from a guinea pig fed HFHC diet for an extended period of 6 months. Degenerative changes are present in enlarged islets, indicated by deposition of fibrous connective tissue (arrow). Scale bar: 100 µm. (E) Islet morphology 3 weeks after STZ treatment in an HFHC/STZ guinea pig. Islets are reduced in size after treatment with STZ (arrow). Scale bar: 200 µm. (F) Immunofluorescent detection of pro-insulin (green) in an enlarged islet from an HFHC-fed guinea pig after 8 weeks of consuming the diet. Enlarged islets contain a high frequency of insulin-producing β cells. Blue is Hoechst nuclear counterstain. Scale bar: 100 µm. (G) Immunofluorescent detection of pro-insulin in an islet from an HFHC/STZ guinea pig 3 weeks after STZ treatment. There is an overall reduction in the frequency of insulin-producing β cells 3 weeks after STZ treatment. Scale bar: 100 µm.

The pancreatic islets of HFHC-fed guinea pigs were, in general, larger in size and quantitatively more numerous, having a mean number of 1034±548.4 /mm^2^ of total evaluated pancreatic tissue after 8 weeks of consuming the HFHC diet, compared with normal-diet controls, which had a mean number of 423.3±186.8 /mm^2^ (Fig. 5A-C). In HFHC/STZ guinea pigs, 21 days post-STZ treatment, pancreatic islets were reduced in size and had indiscernible, irregular margins (Fig. 5E). Immunofluorescent detection of pro-insulin in the HFHC-fed guinea pigs revealed a high frequency of insulin-producing cells in all islets, including those that were significantly enlarged (Fig. 5F). By contrast, immunofluorescent detection of pro-insulin in the HFHC/STZ guinea pigs 21 days post-STZ treatment revealed persistence of insulin-producing β cells, but these were significantly reduced in frequency compared with the normal-diet controls and HFHC-fed guinea pigs (Fig. 5G).

### HFHC/STZ-treated guinea pigs are non-insulin dependent and responsive to oral antihyperglycemic therapy

**Fig. 6.**
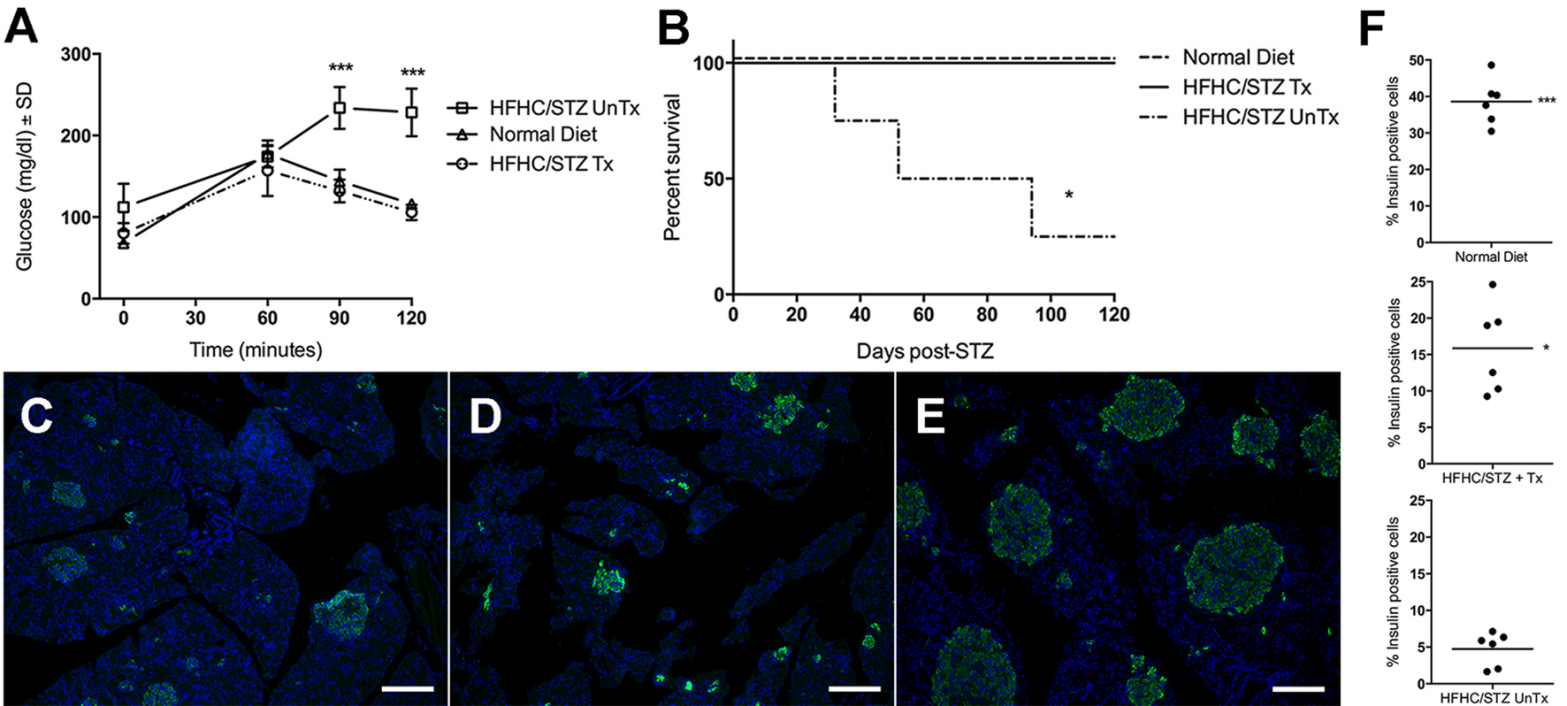
**HFHC/STZ guinea pigs develop insulin-independent diabetes that is responsive to oral antihyperglycemic therapy.** (A) Diabetic HFHC/STZ guinea pigs received either a combination of metformin and glipizide therapy (HFHC/STZ Tx, *n*=4), initiated 21 days after STZ treatment, or remained with uncontrolled diabetes (HFHC/STZ UnTx, *n*=4). Responses to an oral glucose challenge were compared with normal-diet controls (*n*=2). Metformin and glipizide treatment in HFHC/STZ guinea pigs reverses diabetic glucose intolerance and fasting hyperglycemia. ***P<0.001, one-way ANOVA. (B) Survival was tracked for 120 days in HFHC/STZ guinea pigs that were either treated with metformin and glipizide or remained with uncontrolled diabetes, compared with normal-diet control guinea pigs. Antihyperglycemic therapy significantly improves survival in guinea pigs with HFHC/STZ diabetes. *P<0.05. (C) Immunofluorescent detection of pro-insulin after 120 days of treatment in HFHC/STZ guinea pigs treated with metformin and glipizide combination therapy. Reduced β-cell frequency remains present after 120 days in the presence of combination therapy. (D) Immunofluorescent detection of pro-insulin in HFHC/STZ guinea pigs with uncontrolled diabetes. Insulin production appears similar to those receiving combination therapy. (E) Immunofluorescent detection of pro-insulin in a normal-diet control guinea pig from the survival group, demonstrating a greater frequency of β cells in the absence of diabetogenic treatment. (F) Quantitation of pro-insulin-positive cells expressed as a percentage of tissue area from six representative fields per treatment group. HFHC/STZ treatment reduces total β-cell mass, which increases in guinea pigs treated with metformin and glipizide. *P<0.05, ***P<0.001, compared with untreated HFHC/STZ guinea pigs, one-way ANOVA. Scale bars: 100 μm.

In order to confirm a state of type 2 diabetes in HFHC/STZ guinea pigs, the response to the oral antihyperglycemic drugs metformin and glipizide was evaluated. Before initiation of therapy, HFHC/STZ guinea pigs had confirmed glucose intolerance consistent with overt diabetes after 11 weeks in total of HFHC feeding and 21 days post-STZ treatment. Complete reversal of diabetes-related glucose intolerance was evident in HFHC/STZ guinea pigs after 14 days of treatment with a combination of metformin and glipizide (Fig. 6A). Guinea pigs treated with metformin and glipizide in combination displayed 100% survival to 120 days, whereas only a 25% survival rate was evident among mock-treated diabetic controls over the 120-day evaluation period (Fig. 6B). Immunofluorescent detection of insulin-producing β cells in the pancreas of normal-diet controls, untreated HFHC/STZ guinea pigs and HFHC/STZ guinea pigs receiving combination therapy demonstrated that, compared with nondiabetic normal-diet controls, there is a persistent reduction in overall β-cell frequency up to 120 days post-STZ treatment in HFHC/STZ guinea pigs, regardless of whether the guinea pigs received treatment with metformin and glipizide (Fig. 6C-E). However, the frequency of insulin-producing β cells is greater in HFHC/STZ guinea pigs treated with oral antihyperglycemic therapy.

## DISCUSSION

In this study, we developed a model of type 2 diabetes in the guinea pig through a combination of diet-induced glucose intolerance and subtotal β-cell cytotoxicity. In the development of this model, we have confirmed that, similar to the rat and mouse models of diet-induced obesity, feeding a HFHC diet results in glucose intolerance in the guinea pig and compensatory responses that are consistent with insulin resistance, features which mimic a prediabetic state in humans. Moreover, we have identified crucial factors necessary for improved survival, induction of hyperglycemia and sustained diabetic glucose intolerance using a combination of HFHC diet and STZ in the guinea pig to yield a reproducible model of insulin-independent overt type 2 diabetes.

The use of high-fat diets in combination with chemical induction of diabetes has gained recent traction for generation of rodent models of diabetes, particularly to be used in therapeutic testing. Although predominantly reported in the rat, this same strategy has also been used in mouse models but never previously evaluated in the guinea pig. There are advantages to this approach, because this replicates important features of human type 2 diabetes, namely the transition from a prediabetic state to overt diabetes, which requires the loss of functional β-cell mass. Thus, this model of diabetes induction is reflective of prototypical type 2 diabetes because it combines insulin resistance with reduced overall β-cell mass, in a context where impaired systemic responsiveness to insulin is the limiting factor in the failure to control blood glucose. Thus, improving systemic insulin sensitivity is effective in controlling diabetes in this model, without a requirement for insulin-replacement therapy. This insulin-independent phenotype is a defining feature of this guinea-pig model, which has also been previously reported on multiple occasions in rats. This model is in stark contrast to chemical induction of diabetes with STZ alone, which effectively eliminates β-cell populations, leading to an insulin-dependent phenotype and requirement for insulin therapy to maintain glucose tolerance. We show here that STZ alone is relatively ineffective in the guinea-pig model, because this does not produce lasting hyperglycemia or diabetic glucose intolerance even when substantial β-cell mass is eliminated. This might be explained by the ability of guinea pigs to readily compensate for lowered β-cell mass, because β-cell function appears to be intact in response to glucose stimulation, and β-cell mass is improved with antihyperglycemic therapy. Thus, a crucial feature of this combined HFHC-diet and STZ model is the requirement for systemic insulin resistance, which is maintained by the HFHC diet, thereby limiting β-cell compensation in the time frame evaluated in this study.

Guinea pigs fed an HFHC diet demonstrate delayed glucose clearance in response to oral glucose challenge, impaired glucose-lowering effects of exogenous insulin treatment and high fasting serum insulin concentrations, all of which are consistent with systemic insulin resistance. Hyperinsulinemia in the face of normal fasting glucose indicates that these guinea pigs mount a successful β-cell compensatory response to HFHC-induced glucose intolerance, which is further supported by the development of islet hyperplasia in guinea pigs fed the HFHC diet, a typical response of most rodent models in the face of insulin resistance and, to a lesser extent, of humans (Brüning et al., 1997; Butler et al., 2003; Rahier et al., 2008; Jones et al., 2010; Kitamura, 2013). Additionally, functional compensation by β cells was evident in HFHC-fed guinea pigs, with increased insulin production to maintain euglycemia. We found that in this model, a stable diabetic state and decompensated response can be achieved through STZ treatment only after diet-induced glucose intolerance. These data suggest that the metabolic impacts of HFHC diet are sufficient to overcome the compensatory capacity of β cells after STZ exposure, a response that is known to be reduced in overt type 2 diabetic humans (Butler et al., 2007) and is similar to the insulin-resistant rat models, where hyperglycemia results from low doses of STZ that would otherwise have no impact on glucose metabolism in normal wild-type rats (Reaven and Ho, 1991). Data from this study support a strong β-cell compensatory response in the guinea pig, making this a potentially valuable model for studying the factors that influence β-cell regeneration and function in the presence of systemic insulin resistance.

Although guinea pigs fed the HFHC diet did not gain weight at a rate significantly greater than normal-diet controls, the diet did alter lipid metabolism and lead to dyslipidemia when measured after a period of fasting. HFHC feeding promoted high concentrations of triglycerides and increased LDL/VLDL cholesterol, which were increased along with free fatty acids in the serum of HFHC/STZ guinea pigs, features that suggest altered hepatic lipid metabolism and increased lipolysis, both of which are features of insulin resistance in adipose tissue and liver. It has been suggested that one of the greatest contributors to the development of insulin resistance is the accumulation of fat within hepatocytes. Consistent with this finding in human patients, our guinea-pig model also accumulated triglycerides in the liver (Lim et al., 2011). Moreover, this lipid accumulation was reversible with the use of insulin-sensitizing and secretagogue therapy, provided in the form of the biguanide metformin and the sulfonylurea glipizide, consistent with a direct link between systemic insulin resistance and the accumulation of hepatic lipid, as has been demonstrated in human patients with type 2 diabetes (Mazza et al., 2012; Taylor, 2013).

Based on the responses of this guinea-pig model to the HFHC diet, we show that impaired glucose tolerance from feeding the HFHC diet before low-dose STZ treatment contributes significantly to the maintenance of stable diabetic glucose intolerance. In this model, insulin-dependent diabetes was not achieved solely through STZ administration, as is seen in the rat and mouse, where STZ reproducibly leads to β-cell depletion and a state of insulin-deficient diabetes. By contrast, we have shown that in HFHC/STZ guinea pigs insulin production is retained in residual β cells and that fasting serum insulin circulates at concentrations comparable to those of normal-diet controls. This is further supported by sustained glucose-stimulated insulin secretion in HFHC/STZ guinea pigs. Although first- and second-phase insulin secretion cannot be distinguished without clamp techniques or isolated islets, these data suggest that β-cell function remains in HFHC/STZ guinea pigs, which further supports impaired tissue sensitivity to insulin as a major contributor to sustained glucose intolerance in this model. Sustained fasting hyperglycemia was evident only after the compensatory β-cell hyperplasia and hyperinsulinemia of HFHC-fed guinea pigs was abolished through single-dose STZ treatment. Therefore, the compensatory adaptation of insulin-producing β cells in guinea pigs fed a HFHC diet is a crucial factor in the manifestation of overt insulin-independent diabetes after STZ treatment. These findings are similar to studies in human type 2 diabetes patients, where dietary restrictions combined with exercise can alleviate clinical diabetes, indicating that residual β-cell function is sufficient to maintain blood glucose homeostasis once peripheral tissue insulin resistance is resolved (Taylor, 2013). Although this has not yet been confirmed through experimental glucose-clamp techniques, our data indicate that HFHC/STZ guinea pigs develop overt diabetes largely as a result of insulin resistance, which is similar to the insulin-independent state typical of human patients with type 2 diabetes. Although HFHC/STZ guinea pigs have reduced β-cell mass, residual function is present based on glucose-stimulated insulin secretion, supporting the requirement for insulin resistance to overcome the insulin compensation that occurs even in the presence of limited remaining β-cell mass. In further support of an insulin-independent diabetic state in this model, we have demonstrated that HFHC/STZ guinea pigs not only respond clinically to a combination of metformin and glipizide with reversal of glucose intolerance, similar to a rat model, but also that this drug combination significantly improves survival without the need for exogenous insulin therapy, which is routinely required for survival in other rodent models with STZ-induced diabetes (Haughton et al., 1999; Reed et al., 2000). Moreover, guinea pigs treated with this combination therapy had improved β-cell mass, suggesting that control of tissue sensitivity affords some level of β-cell protection.

In contrast to our guinea-pig model, the use of STZ to establish insulin-dependent hyperglycemia in mice and rats is well established. The protocols for use of STZ in these rodents, compared with what has been identified in our guinea-pig model, demonstrate the wide variability in the response of multiple rodent species to STZ. The rat model requires only a single low dose of STZ for effective β-cell cytoxicity, whereas five consecutive doses of 50 mg/kg are generally accepted as the most effective dosing schedule for mice (Lenzen, 2007, 2008). Comparatively, we demonstrate here that the guinea pig generally responds more favorably to higher doses of STZ as a single bolus; however, there is a narrow toxic threshold, and hyperglycemia is not sustained. This study indicates that high mortality is associated with a cumulative STZ dose of 300 mg/kg, but doses of 100 mg/kg fail to elicit hyperglycemia in guinea pigs. Paradoxically, the lowest frequency of hyperglycemia was noted with the highest STZ dose of 300 mg/kg, whereas mid-range doses of 200 and 250 mg/kg yielded hyperglycemia at varying levels. This is further supported by a lack of histological evidence of β-cell death in guinea pigs receiving the 300 mg/kg dose (data not shown). This could be explained by the level of acute toxicity seen at this dose, which precluded the establishment of diabetes because of failure to target β cells selectively for cytotoxicity (Lenzen, 2007). Additionally, the rapid clinical decline and mortality might have occurred at a rate preceding diabetogenic effects detectable by random blood glucose sampling. A lack of β-cell specificity at the high 300 mg/kg dose is confirmed by pathological lesions in guinea pigs suffering early mortality post-STZ treatment that were consistent with acute STZ-induced toxicity of other major cell types expressing the GLUT2 solute transporter. GLUT2 is the cellular glucose transporter isoform previously linked to tissue specificity of STZ and cytotoxic effects on β cells, but is also expressed by intestinal epithelium, kidney tubular epithelium and hepatocytes (Bell et al., 1990; Schnedl et al., 1994; Rees and Alcolado, 2005). By contrast, at doses capable of inducing hyperglycemia, STZ administration did lead to specific β-cell death, which supports previous evidence that guinea-pig β cells express the GLUT2 solute transporter, similar to other rodent species (Hosokawa et al., 2001; Lenzen, 2007).

Although dose appears to impact STZ tolerance and the development of hyperglycemia in the guinea pig, we have also demonstrated similar efficacy of STZ solution whether administered as a freshly prepared solution or after allowing for anomer equilibration. It is frequently suggested that the unstable α-anomer of STZ is more diabetogenic than the β-anomer and, for this reason, it is often cited that STZ must be prepared fresh and administered immediately (Hegde et al., 2003; Ceylan-Isik et al., 2006; Gurley et al., 2006; Lenzen, 2007). However, more recently it has been demonstrated that a stable equilibrium is established between α- and β-anomeric forms of STZ within 2 h of dissolution, and this preparation leads to consistent hyperglycemia in mice, with a significant reduction in mortality (de la Garza-Rodea et al., 2010). These findings are recapitulated in our guinea-pig model, which demonstrates that anomer-equilibrated STZ is equally effective in inducing hyperglycemia in guinea pigs compared with an equivalent dose of freshly prepared STZ. This study therefore provides additional support for the adoption of anomer equilibration in STZ chemical-induced animal models of diabetes in an attempt to improve reproducibility and reliability of studies between laboratories.

In line with previously reported resistance of guinea pigs to STZ, we experienced a rate of diabetes induction at doses of 200 and 250 mg/kg that was both variable and incomplete (Kushner et al., 1969; Losert et al., 1971; Lenzen, 2007; Aslan et al., 2013). Previously, it was reported that guinea pigs were largely insensitive to the cytotoxic effects of STZ or of alloxan, an alternative GLUT2-dependent β-cell toxin, when administered systemically; however, administration directly into the pancreatic circulation yielded uniform hyperglycemia from alloxan, and isolated guinea-pig β cells are susceptible to *ex vivo* STZ treatment (Lenzen, 2007, 2008). Thus, the reported level of resistance to both alloxan and STZ in the guinea pig might not be a direct reflection of the β cell itself, but rather the availability of the compound to the β cells after systemic administration. This is further supported by the fact that isolated pancreatic islets from the guinea pig are sensitive to the actions of both compounds *in vitro* (Griffiths, 1948; Lenzen, 2007). We were able to improve upon the response to systemic STZ administration through the use of the α_2_-adrenergic receptor antagonist yohimbine, before administering STZ, which resulted in selective β-cell death in guinea pigs analyzed within 48 h of STZ administration. It has been previously demonstrated that STZ-induced diabetes could be improved through the use of a specific α_2_-adrenergic receptor antagonist but not through blockade of α_1_ receptors (Nakadate et al., 1981; lsner et al., 2000; Rosengren et al., 2010). The observation that increased circulating insulin coincides with enhanced STZ efficacy in mice and rats suggests that the mechanism of this synergistic effect might revolve around preventing catecholamine–adrenergic receptor signaling, which is known to inhibit insulin release from β cells (Jansson and Sandler, 1985; Nakadate et al., 1981; Ar'Rajab and Ahrén, 1991; Savontaus et al., 2008). However, it remains to be determined whether the role of insulin secretion in yohimbine sensitization to STZ is attributable to enhanced uptake of the STZ glucose analogue or downstream intracellular actions within the β cell.

Regardless of increased STZ efficacy when combined with yohimbine, we continued to observe steadily decreasing blood glucose and an improved response to oral glucose challenge over time in STZ-treated normal-diet guinea pigs, indicating spontaneous recovery from diabetes induced with STZ alone. Multiple studies have used guinea pigs with STZ-induced diabetes, carried out for up to 4 weeks after STZ administration; however, the rate of fasting and nonfasting hyperglycemia, as well as response to glucose challenge over time after STZ treatment have not been well documented (Hootman et al., 1998; LePard, 2005; Aslan et al., 2013; Xie et al., 2013; Saidullah et al., 2014; Tocchetti et al., 2015). Therefore, it is uncertain how often spontaneous recovery is observed in the guinea pigs, although this recovery is recognized in STZ-treated rats, with β-cell regeneration evident within 7 days of treatment (Movassat et al., 1997; Garofano et al., 2000). The improvement in glucose tolerance over time observed in the development of this model suggests that residual β-cell compensation, with or without regeneration, might overcome the initial effects of STZ for the dose, method of preparation and route of administration used in this study. Moreover, the data in this study support an increase in islet β-cell mass over an extended period after STZ administration, indicating a regenerative response in STZ-treated guinea pigs. However, this is insufficient to overcome the diet-induced glucose intolerance in HFHC-fed guinea pigs.

Altogether, the guinea-pig model of type 2 diabetes developed in this study closely mimics the classical pathogenesis of type 2 diabetes, where hyperinsulinemic compensation for insulin resistance is ultimately lost through diminished β-cell capacity. Although this guinea-pig model might be applicable for certain experimental conditions, including comorbidities, contributions of dyslipidemia and compensatory or regenerative β-cell responses, this model is not expected to replace other rodent models and is not without limitations. The generation of reproducible diabetic guinea pigs using this model system requires intensive effort, exceeding that required to induce diabetes in more simple rodent models of high-fat diet or STZ alone; however, those models do not replicate the same features of human type 2 diabetes that are seen in this guinea-pig model. The important similarities between this model and human type 2 diabetes, including glucose intolerance that precedes diabetic hyperglycemia, altered lipid metabolism, reduced compensatory β-cell capacity and responsiveness to oral antihyperglycemic therapy, will significantly enhance the use of the guinea pig as a model for type 2 diabetes research.

## MATERIALS AND METHODS

### Animals and sample collection

Female, outbred Dunkin–Hartley guinea pigs, weighing between 250 and 300 g, were purchased from Charles River Laboratories or inbred Strain-13 guinea pigs were bred at the Colorado State University Laboratory Animal Resources facility, and diabetogenic treatments were initiated at a weight of ∼300 g in guinea pigs of either sex. For collection of serum, guinea pigs were anesthetized via isoflurane inhalation and blood was collected by percutaneous venipuncture of the cranial vena cava. Blood glucose was measured using the Freestyle Lite glucometer (Abbot, Alameda, CA, USA) from a skin-prick site adjacent to the most peripheral vein on the pinna, previously validated against the glucose oxidase enzymatic assay. At the time of euthanasia, guinea pigs were administered 40 mg of ketamine and 0.5 mg of diazepam via intramusclar injection for anesthetic induction. Anesthetized guinea pigs were administered a 750 mg dose of pentobarbital via the IP route for euthanasia. All animal experiments were performed in accordance with the National Research Council's Guide for the Care and Use of Laboratory Animals and were approved by the Animal Care and Usage Committee at Colorado State University under protocol number 10-1990A.

### Preparation of STZ solution for injection

All STZ preparations were prepared as 100 mg/ml solutions in sodium citrate buffer at a pH of 4.5. Sodium citrate buffer for injection was prepared initially as a 40 mM citric acid solution (pH 2.9). The citric acid solution was titrated with 40 mM sodium citrate until a pH of 4.5 was reached. STZ, >75% α-anomer purity (Sigma, St Louis, MO, USA), was dissolved in the freshly prepared sodium citrate buffer at a concentration of 100 mg/ml. The STZ solution was passed through a 0.22 µm filter to sterilize, and administered either freshly prepared or after equilibration for 2 h at 4°C and protected from light.

### Optimization of STZ treatment for induction of diabetic hyperglycemia

Various doses of STZ and routes of administration were evaluated for diabetogenic efficacy and level of mortality in guinea pigs initially fed a normal diet and, subsequently, in guinea pigs with diet-induced impaired glucose tolerance. Guinea pigs were administered STZ via the SC or IP route, at doses of 300, 250, 200 or 100 mg/kg or as multiple daily consecutive doses of 50 mg/kg for 4 or 6 days. In a separate experiment, fresh or anomer-equilibrated STZ was administered via the SC route at a dose of 200 mg/kg. Additionally, the diabetogenic efficacy of STZ was investigated for enhancement by pretreating guinea pigs with 0.5 mg/kg of yohimbine, an α_2_-adrenergic receptor antagonist, by intramuscular injection 20-30 min before administration of STZ. For the depletion of β-cell mass in HFHC-fed guinea pigs, individuals with evidence of impaired glucose tolerance were administered a single 200 mg/kg dose of anomer-equilibrated STZ after pretreatment with 0.5 mg/kg yohimbine.

### Custom diet formulation

Guinea pigs were fed a custom-formulated HFHC diet (Dyets Inc., Bethlehem, PA, USA). The custom-formulated diet consisted of total calories as 30% fat, 52% carbohydrate and 18% protein, where calories from fat were derived equally from Primex vegetable shortening and beef tallow, and carbohydrate calories were composed of 55% fructose and 45% sucrose. The fatty acid composition of the HFHC diet consisted of 41.9% saturated, 50.3% monounsaturated and 7.8% polyunsaturated fatty acids. For comparison, control guinea pigs with normal glucose tolerance were fed a commercially available conventional guinea-pig diet containing 3% fat, 18% protein and 55% complex carbohydrates derived from grain (Harlan-Teklad 2040).

### Evaluation of impaired glucose tolerance and response to insulin

To evaluate the glucose-lowering effects of insulin, guinea pigs fed the normal diet or the HFHC diet for 4 or 8 weeks or the HFHC for 8 weeks followed by STZ treatement were administered an SC injection of 0.5 units/kg of regular-acting human recombinant insulin (Humulin-R; Eli Lilly, Indianapolis, IN, USA). At the time of injection, glucose was measured in nonfasted guinea pigs using the Freestyle Lite glucometer, then measured at 25, 50, 75 and 100 min after administration. To determine glucose tolerance in normal-diet controls, guinea pigs fed HFHC diet (HFHC-fed) or guinea pigs rendered diabetic by HFHC feeding and STZ treatment (HFHC/STZ), a standardized oral glucose challenge consisting of a 2 g/kg bolus of d-glucose (0.5 g/ml) was administered after a 12 h fasting period (OGTT). Glucose concentrations were measured at times 0, 60, 90, 120 or 150 min post-administration with a Freestyle Lite glucometer, validated for accuracy against the glucose oxidase method for quantification of glucose in serum.

### Quantification of lipid parameters

Total serum free fatty acid concentrations were measured by fluorescent detection of hydrogen peroxide generated by oxidation of acyl CoA (Cayman Chemical, Ann Arbor, MI, USA). Total serum triglycerides were quantified spectrophotometrically after sequential enzymatic conversion with lipoprotein lipase, glycerol kinase and glycerol phosphate oxidase followed by peroxidase-mediated colorimetric change (Cayman Chemical), as previously described (Podell et al., 2012). For measurement of total hepatocellular triglycerides, ∼400 mg of liver tissue was homogenized in assay buffer containing protease inhibitor cocktail (Thermo Scientific, Waltham, MA, USA), centrifuged at 15,000 *g*, and triglycerides were measured in supernatant using the enzymatic assay. Calculated liver triglyceride concentrations were normalized to the total protein in the supernatant, as measured by BCA assay (Thermo Scientific). Total, high-density lipoprotein (HDL) and LDL cholesterol was quantified in serum samples from normal-diet, HFHC-fed and HFHC/STZ guinea pigs from two separate experiments (total *n*=10 per group). Using the HDL and LDL/VLDL cholesterol assay (Cell BioLabs, San Diego, CA, USA), LDL and VLDL particles were precipitated from serum samples, and cholesterol was quantified in supernatant and precipitated fractions to determine the cholesterol content of HDL and LDL/VLDL particles, respectively.

### Quantification of serum insulin

Owing to well-documented divergence of the guinea-pig insulin sequence and, thereby, lack of commercially available cross-reactive immunoassays, a direct enzyme-linked immunosorbent assay (ELISA) for detection of guinea-pig insulin was developed based on the previously described guinea-pig insulin peptide immunogen (Chan et al., 1984; Maimon et al., 1991). The C-terminal decapeptide of the guinea-pig insulin β chain, amino acid sequence DDGFFYIPKD (Maimon et al., 1991), was conjugated to the C-terminus of bovine serum albumin (Bio-Synthesis Inc., Lewisville, TX, USA). Four Balb/C mice were immunized with 100 µg of this conjugated protein initially in TiterMax adjuvant (Sigma), then twice additionally at 3 week intervals in Freund's incomplete adjuvant (Sigma). Serum from each mouse was evaluated for confirmation of specific antibody by direct ELISA before euthanasia, and specific reactivity was confirmed by specific localized binding to islet cells in guinea-pig pancreas by immunofluorescent staining. For the detection of guinea-pig insulin, blood was collected from fasted guinea pigs, corresponding fasting glucose concentrations measured, and 30 kDa filtered serum was coated on high-binding polystyrene plates overnight at 4°C at a 1:10 dilution in sodium bicarbonate buffer (pH 9.0). Guinea-pig insulin was detected with pooled polyclonal mouse antiserum (1:1000), followed by horseradish peroxidase goat anti-mouse IgG antibody (1:1000) and 3,3,5,5-tetramethylbenzidine substrate, and concentration calculated based on a standard curve ranging from 0.1 to 7 ng/ml of insulin. Serum from unimmunized mice was used as a negative control.

### Measurement of glucose-stimulated insulin secretion

Guinea pigs fed a normal diet, an HFHC diet for 8 weeks or combined HFHC diet for 8 weeks with subsequent STZ treatment were subjected to oral glucose challenge as described for the OGTT. After the 12 h fasting period, blood was collected for quantification of fasting insulin, then guinea pigs were fed the 2 g/kg glucose bolus. After 30 min, repeated blood collection was performed for quantification of insulin secretion stimulated by an oral glucose challenge. Insulin was quantified in serum by direct ELISA.

### Treatment with oral antihyperglycemic therapy

Beginning at 3 weeks after STZ treatment, diabetic guinea pigs were treated with water suspensions of either metformin alone (25 mg) or with glipizide (0.25 mg) in combination, administered daily *per os*. The efficacy of treatment was assessed after 14 days of daily therapy based on improvement in glucose tolerance by OGTT. Normal-diet controls and HFHC/STZ guinea pigs, either untreated or treated with the metformin and glipizide combination therapy, were followed for survival until 120 days after initiation of oral therapy.

### Histology and area morphometry of guinea-pig pancreas

Sections of pancreas were sampled from either end of the pancreatic limbs and at the center of the organ at the time of necropsy and fixed in 4% buffered paraformaldehyde. The tissues were paraffin embedded and 5-µm sections stained with hematoxylin and eosin using routine methods for histopathological evaluation. Morphometric analysis was performed using a Nikon 80i Eclipse microscope and StereoInvestigator software, version 10.02 (MBF Bioscience, Williston, VT, USA), with tissue area estimated using the area fraction fractionator method, as previously described (Ordway et al., 2010). The frequencies of islets within the quantified area were counted to determine a ratio of islets per millimeter squared of pancreatic tissue.

### Immunofluorescent detection and quantification of pro-insulin in pancreatic tissue

Paraffin-embedded 5 µm sections of pancreas were deparaffinized and rehydrated in serial ethanol washes followed by antigen retrieval by boiling in target retrieval buffer (Dako, Carpinteria, CA, USA) for 30 min. Endogenous peroxidase activity was quenched with 0.3% H_2_O_2_ and blocking performed in 0.15 mM glycine and 10% fetal bovine serum, 1% bovine serum albumin in PBS. Tissue sections were incubated with anti-pro-insulin monoclonal antibody, clone K36AC10 (Abcam, ab6995), at a 1:500 dilution overnight at 4°C. After three washes in Tween-TBS, tissue sections were incubated with horseradish peroxidase-conjugated anti-mouse IgG at 1:500 dilution (Vector Laboratories, PI-2000) for 60 min at room temperature, washed three times in Tween-TBS, and the signal amplified with the tyramide signal amplification kit (Life Technologies, Grand Island, NY, USA) using Alexa Fluor 488-labeled tyramide, as directed by the manufacturer. Purified murine IgG1 was used as an isotype control. The frequency of pro-insulin detection by immunofluorescence was quantified by image analysis using Nikon NIS Elements software. Background fluorescence was subtracted based on mouse IgG1 isotype controls and intensity thresholds set for detection of green (pro-insulin) and blue (Hoechst nuclear stain). Frequency of pro-insulin immunoreactivity was normalized to the number of cells based on the frequency of Hoechst staining and expressed as a percentage ratio.

### Data analysis

Sample size was defined by power analysis based on the difference in calculated area under the curve for OGTT performed on nondiabetic and HFHC/STZ guinea pigs receiving the diabetogenic protocol described above with α set at 0.05. With a sample size of three animals per group and equivalent subjects to controls, there is an 80% probability of detecting a true mean difference of at least 5539 glucose×minutes (power of 0.8). No animals were excluded from the statistical analyses. Allocation to experimental groups was randomly determined by animal care staff at the time of animal arrival and cage assignment. All quantitative data are expressed as means±s.d. for descriptive statistical representation or, where data are represented as individual points, the line represents the group mean. Statistical analyses and graphic expression of data were performed using GraphPad Prism, version 6 and SAS version 9.3. Comparison of data between treatment groups was performed using Student's unpaired *t*-test or two-way ANOVA followed by *post-hoc* test for pair-wise comparison of means, with significance set at *P*≤0.05.
